# Omega-3 Polyunsaturated Fatty Acids Suppress the Cystic Lesion Formation of Peritoneal Endometriosis in Transgenic Mouse Models

**DOI:** 10.1371/journal.pone.0073085

**Published:** 2013-09-10

**Authors:** Kensuke Tomio, Kei Kawana, Ayumi Taguchi, Yosuke Isobe, Ryo Iwamoto, Aki Yamashita, Satoko Kojima, Mayuyo Mori, Takeshi Nagamatsu, Takahide Arimoto, Katsutoshi Oda, Yutaka Osuga, Yuji Taketani, Jing X. Kang, Hiroyuki Arai, Makoto Arita, Shiro Kozuma, Tomoyuki Fujii

**Affiliations:** 1 Department of Obstetrics and Gynecology, Faculty of Medicine, The University of Tokyo, Tokyo, Japan; 2 Department of Health Chemistry, Graduate School of Pharmaceutical Sciences, The University of Tokyo, Tokyo, Japan; 3 Department of Medicine, Massachusetts General Hospital and Harvard Medical School, Boston, Massachusetts, United States of America; Gentofte University Hospital, Denmark

## Abstract

Omega-3 polyunsaturated fatty acids (omega-3 PUFAs) play a role in controlling pathological inflammatory reactions. Endometriosis is characterized by the presence of endometrial tissue on the peritoneum and an exaggerated inflammatory environment around ectopic tissues. Here peritoneal endometriosis was reproduced using a mouse model in which murine endometrial fragments were inoculated into the peritoneal cavity of mice. Fat-1 mice, in which omega-6 can be converted to omega-3 PUFAs, or wild type mice, in which it cannot, were used for the endometriosis model to address the actions of omega-3 PUFAs on the development of endometriotic lesions. The number and weight of cystic endometriotic lesions in fat-1 mice two weeks after inoculation were significantly less than half to those of controls. Mediator lipidomics revealed that cystic endometriotic lesions and peritoneal fluids were abundant in 12/15-hydroxyeicosapentaenoic acid (12/15-HEPE), derived from eicosapentaenoic acid (EPA), and their amount in fat-1 mice was significantly larger than that in controls. 12/15-Lipoxygenase (12/15-LOX)-knockout (KO) and control mice with or without EPA administration were assessed for the endometriosis model. EPA administration decreased the number of lesions in controls but not in 12/15-LOX-KO mice. The peritoneal fluids in EPA-fed 12/15-LOX-KO mice contained reduced levels of EPA metabolites such as 12/15-HEPE and EPA-derived resolvin E3 even after EPA administration. cDNA microarrays of endometriotic lesions revealed that Interleukin-6 (IL-6) expression in fat-1 mice was significantly lower than that in controls. These results suggest that both endogenous and exogenous EPA-derived PUFAs protect against the development of endometriosis through their anti-inflammatory effects and, in particular, the 12/15-LOX-pathway products of EPA may be key mediators to suppress endometriosis.

## Introduction

Eicosapentaenoic acid (EPA, C20:5n-3) and Docosahexaenoic acid (DHA, C22:6n-3) are representative omega-3 polyunsaturated fatty acids (PUFAs) and show anti-inflammatory effects in acute and chronic inflammatory conditions [1]. Increased levels of omega-3 PUFAs in a tissue lead to the formation of functional EPA or DHA-derived mediators, such as resolvins and protectins, and protects from tissue damage [2]. Many studies have been focused on their effect in resolving inflammation mostly through reductions in neutrophil trafficking and upregulation of macrophage-mediated removal of apoptotic cells [3]. This is associated with attenuated pro-inflammatory signaling by Leukotriene B4 Receptor 1 (BLT1) and ChemR23, which are expressed on neutrophils and macrophages [4], [5], respectively, and decreased activity of the pro-inflammatory transcription factor nuclear factor kappa beta (NF-κB) [6], [7].

Dietary supplementation is a traditional approach to modify tissue nutrient composition in animal studies of nutrition. Feeding animals different diets that consist of many components derived from different materials can cause many variations between experimental groups. Kang et al. recently engineered a transgenic mouse that carries the fat-1 gene from the roundworm *Caenorhabditis elegans* [8]. This gene encodes an omega-3 fatty acid desaturase that catalyzes conversion of omega-6 to omega-3 PUFAs and that is absent in most animals, including mammals. There is a remarkable difference in the tissue omega-6/omega-3 PUFA ratio between wild type and fat-1 transgenic mice [9]. Fat-1 mice, which can have an altered ratio of omega-6 to omega-3 PUFAs in their tissues and organs independent of diet, allow carefully controlled studies to be performed in the absence of potential confounding factors of diet and, therefore, are a useful model to investigate the biological properties of endogenous omega-3 PUFAs [8].

Endometriosis is one of the most common gynecological disorders and is a chronic condition characterized by the presence of endometrial tissue outside the uterine cavity, most commonly on the pelvic peritoneum and ovaries. The disease can involve adjacent organs such as the fallopian tubes, bladder, and recto-sigmoid colon. Endometriosis has a prevalence of up to 50% among infertile women and is associated with various distressing symptoms, including dysmenorrhea, pelvic pain, and infertility [10]. These symptoms are thought to be the result of an excessive inflammatory environment within the peritoneal cavity. Numerous studies support that elevated numbers of activated immune cells, particularly peritoneal macrophages, are involved in the molecular and cellular processes that lead to endometriotic lesion development; initiation, maintenance, and progression of endometriotic lesions [11]. Peritoneal macrophages are known to express inflammatory cytokines, such as IL-6, IL-1β and tumor necrosis factor alpha (TNF-α) [12]–[14] and to be increased in number and more activated in patients with endometriosis [15]. Ectopic endometrial tissues in the peritoneum not only express proinflammatory cytokines in a dysregulated manner, but also elicit aberrant immune influencing factors in the peritoneal fluid, creating a local inflammatory environment [16]. These include prostaglandins (PGs), IL-8 and monocyte chemotactic peptide 1 (MCP-1). IL-8 levels are elevated in the peritoneal fluid of women with endometriosis and levels have been correlated with the severity of the disease [17]. Many studies demonstrated that iron overload originates from lysis of pelvic erythrocytes accumulated by retrograde menstruation and induces oxidative stress in the pelvic cavity. Iron storage levels are higher in the peritoneal macrophages of endometriosis patients than those of controls [18]. Oxidative stress promotes the NF-κB pathway as well as DNA damage. NF-κB-activated macrophages express proinflammatory, growth, and angiogenic factors, such as inducible Nitric Oxide (iNOS), cyclooxygenase-2 (COX-2), IL-1, IL-6, IL-8, TNF-α, and vascular endothelial growth factor (VEGF), which contribute to endometriosis pathogenesis and possible carcinogenesis [19].

Many laboratories have established a variety of experimental animal models of endometriosis, in which endometriotic lesions develop in the peritoneum of small animals, such as the rabbit, rat, and mouse[20]–[24]. These models use homologous or heterologous endometria obtained from congenic animal or human specimens, respectively, as an inoculant into the animal peritoneum. As one of heterologous models, the severe immunodeficient (SCID) mouse is used as a recipient animal and then the model is unsuitable for experiments to investigate immune responses or immunomodulatory effects in endometriotic lesions [25]. We established the homologous model in which the uterus of immunologically normal C57BL/6 strain mice was minced and injected into the peritoneal cavity of the same strain mice [21]. In this model, recipient mice develop endometriotic lesions at the peritoneum, omentum, perivisceral fat tissue, intestinal, and uterine surface. These lesions progress more in mice exposed to estradiol after inoculation of endometrial fragments than that with non-exposed mice, indicating that our model mimics endometriosis in human.

Viewing the important impact of omega-3 PUFAs on pathological inflammatory reactions, we hypothesized that omega-3 PUFAs exhibit a protective action on the chronic inflammatory condition of endometriosis. In this study, fat-1 mice were utilized to address the biological properties of omega-3 PUFAs in the homologous model of endometriosis. We here demonstrated that the endogenous production of omega-3 PUFAs and exogenous EPA affords protection against the development of peritoneal endometriotic lesions.

## Materials and Methods

### Animals and Diets

Fat-1 mice were created on a C57BL/6 background and heterozygote as described [9] and subsequently backcrossed (at least four times) onto a C57BL/6 background. 12/15-LOX-KO mice on a C57BL/6 background were obtained from The Jackson Laboratory (Bar Harbor, ME). Animals were fed a special diet (AIN-76A+10% safflower oil; CLEA Japan, Inc.) that contained 10.3% total fat with fatty acid composition of C16:0 (7.6%), C18:0 (2.7%), C18:1n-9 (14.1%), C18:2n-6 (73.2%), C18: 3n-3 (0.3%), C20:4n-6 (<0.1%), C20:5n-3 (<0.1%), C22: 6n-3 (<0.1%), high in n-6 and low in n-3 fatty acids, until the desired age (6–8 weeks) for experiments. EPA was administered by the addition of 5% EPA ethyl ester (kindly provided by Mochida Pharmaceutical Co., Ltd. Japan) in the fish meal-free F1 diet (Funabashi farm Co., Ltd. Japan) that contained 4.4% total fat with fatty acid composition of C16:0 (14.9%), C18:0 (5.0%), C18:1n-9 (20.8%), C18:2n-6 (52.4%), C18:3n-3 (4.9%), C20:4n-6 (<0.1%), C20:5n-3 (<0.1%), C22:6n-3 (<0.1%). To prevent the oxidation of lipids, all diet was stored in the refrigerator with antioxidants (AGELESS; Mitsubishi Gas Chemical Inc.), and prepared newly every two days. Animal studies were approved by the University of Tokyo Animal Committee.

### Endometriosis model in mice

Both donor and recipient mice, 6–8 weeks old, were ovariectomized to remove the effects of endogenous hormonal changes. All donor mice and recipient mice were injected s.c. with 100 μg/kg estradiol depot (ASKA Pharmaceutical Co., Ltd, Tokyo, Japan) in corn oil every week from the time of the ovariectomy. Two weeks after the ovariectomy, donor mice were killed by cervical dislocation. Uterine horns were removed and put into a dish containing phosphate-buffered saline (PBS). Endometrial fragments, obtained by peeling off the serosa and myometrium gently, were minced using a razor blade. Fragments suspended in 0.6 ml PBS were injected with an 18-gauge needle through the abdominal wall just below the umbilicus into the peritoneal cavity of recipient mice with a ratio of one donor to two recipients. Fat-1, 12/15-LOX-KO, or littermate wild type mice were used as both donor and recipient mice for each group. Four mice for each group were sacrificed two weeks after the injection and peritoneal endometriotic lesions and peritoneal washes were collected.

### Evaluation of peritoneal lesions

Peritoneal lesions forming a cystic lesion were covered with surrounding stromal cells histologically, backed by endometrial cells, and showed much more at the omentum, pelvic cavity, mesenterium and diaphragm. After counting the number of cystic lesions, they were completely excised, fixed with 2% Paraformaldehyd (PFA) + PBS (WAKO, Osaka, Japan), and embedded in paraffin. Serial sections were cut at 8 μm thickness on a cryostat and mounted on poly-L-lysine-treated slides. They were stained with hematoxylin-eosin (HE) and observed under microscopy (BX50; Olympus, Japan). The histological diagnosis of endometriosis was based on the morphological identification of endometriotic glands and stroma. The weight of endometriotic lesions was measured.

### Lipid mediator analyses

LC-MS/MS-based mediator lipidomics was performed as described previously [26]. Briefly, samples were extracted by solid-phase extraction using Sep-Pak C18 cartridges (Waters, Milford, MA, USA) with deuterium-labeled internal standards (PGE_2_-d4, LTB_4_-d4, 15-HETE-d8, arachidonic acid-d8). LC-MS/MS-based lipidomic analyses are performed on Acquity UPLC BEH C_18_ column (1.0 mm×150 mm×1.7 μm) using Acquity UltraPerformance LC system (Waters Co.) coupled to an electrospray (ESI) triple quadrupole mass spectrometer (QTRAP5500; AB SCIEX). The MS/MS analyses were performed in negative ion mode, and the eicosanoids and docosanoids were identified and quantified by multiple reaction monitoring. Calibration curves between 1 and 1000 pg and the LC retention times for each compounds were constructed with synthetic standards.

### cDNA Microarray

We collected cystic lesions 2 weeks after injection of minced endometrium, and total RNA was extracted from their lesions. they were incubated in RNA later. For the cDNA microarray analysis, 0.5 μg of pooled total RNA was amplified and labeled using an Amino Allyl MessageAmpTM II aRNA Amplification kit (Applied Biosystems, Foster City, CA, USA) according to the manufacturer’s instructions. Each sample of aRNA labeled with Cy3 and reference aRNA labeled with Cy5 was cohybridized with GeneTM Mouse Oligo chip 24 k (Toray Industries Inc., Tokyo, Japan) at 37°C for 16 h. After hybridization, each DNA chip was washed and dried. Hybridization signals derived from Cy3 and Cy5 were scanned using Scan Array Express (PerkinElmer, Waltham, MA, USA). The scanned image was analyzed using GenePix Pro (MDS Analytical Technologies, Sunnyvale, CA, USA). All analyzed data were scaled by global normalization.

### RT-PCR for peritoneal macrophages

After washing the peritoneal cavity of each mouse with 5 ml PBS, peritoneal washes were filtered through a 30 μm strainer and centrifuged for 5 min at 200 g. From recovered cellular components, CD11b-positive macrophages were isolated using an isolation kit and incubated in RNA later. We isolated RNA from these macrophages after homogenizing. Two μg of total RNA was extracted using an RNeasy Mini Kit (Qiagen, Hilden, Germany), followed by reverse transcription. We amplified cDNA for 40 cycles with a Light Cycler 480 (Roche, Basel, Switzerland) using a Universal Probe Master and the following primers and Universal Probe Library (UPL) probes (Roche): Interleukin-6 (IL-6) Forward (gctaccaaactggatataatcagga) and Reverse (ccaggtagctatggtactccagaa), with the UPL Probe #6 and β-actin (ACTB) Forward (ctaaggccaaccgtgaaaag) and Reverse (accagaggcatacagggaca), with the UPL Probe #64. We calculated expression levels by the comparative CT method using β-actin as an endogenous reference gene.

### Statistical Analysis

Results were expressed as mean ± standard error. Comparisons between the two groups were performed using the Mann-Whitney U test. Comparisons between multi-groups were performed using the Tukey-Kramer post hoc multiple comparisons test.

## Results

### Comparison of endometriotic lesions between fat-1 and wild type mice

**Figure 1:**
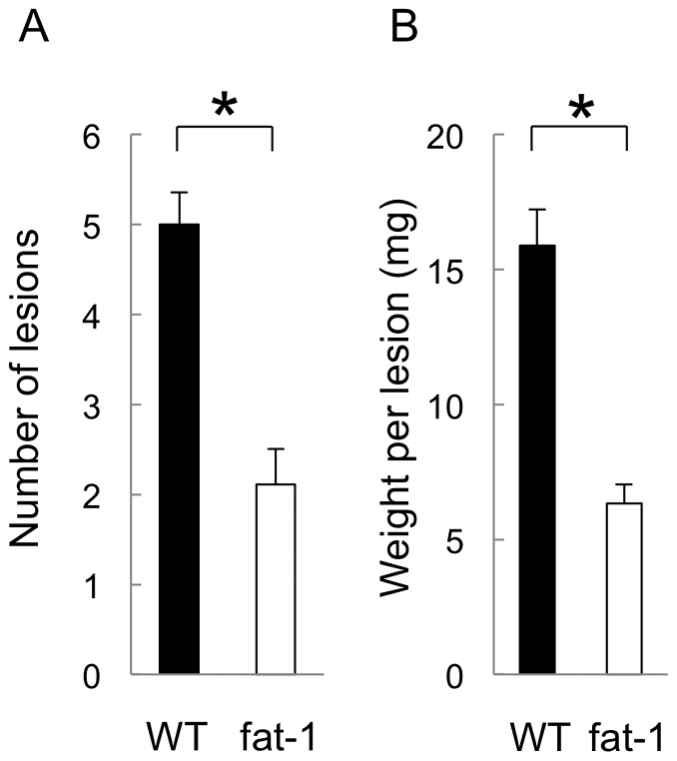
Development of endometriotic lesions in fat-1 and wild type mice. A cystic mass was histologically confirmed as an endometriotic lesion. (A) The number of lesions was counted macroscopically. (B) All masses were resected. The weight (mg) per lesion was measured. These data were compared between the fat-1 and wild type (WT) mice (n = 10 in each group). Mean values with standard deviations are presented. Asterisks indicate those comparisons (fat-1 vs. wild type mice) with statistical significance (p<0.05).

Fat-1 and littermate wild type mice were fed with a diet enriched in omega-6 PUFAs. Omega-6 PUFAs are converted into omega-3 PUFAs by fat-1, *C. elegans* n-3 fatty acid desaturase, in fat-1 mice. The high omega-6 diet led to wide differences in fatty acid profiles, i.e. low (fat-1) vs. high (wild type) omega-6/omega-3 ratios in a litter of mice born to the same mother. We then generated the homologous endometriosis model in which the uterus of fat-1 or wild type mice was minced and injected into the peritoneal cavity of fat-1 or wild type mice, respectively. The AA/EPA + DHA ratio of uterine tissues from the fat-1 mice was 0.82 and from the wild type mice was 2.14. Endometrial fragments were incubated in the peritoneal cavity of mice for two weeks with estrogen treatment. After incubation, mice were sacrificed and the whole peritoneal cavity was examined carefully. Both fat-1 and wild type mice had a scattering of approximately 2–5 mm of cystic mass on the peritoneum. The number of cystic lesions was counted macroscopically and the cystic mass was resected separately for evaluation of the weight. A comparison was made for the number and weight of endometriotic lesions between fat-1 and wild type mice (n = 10 in each group) (Fig. 1). The cystic mass, composed of monolayer columnar epithelia and endometrial stroma, was histologically diagnosed as an endometrial cyst (data not shown). In fat-1 mice, the number of lesions was fewer than half and the weight per lesion was less than half that of wild type mice, indicating that the development of cystic endometriotic lesions were dramatically reduced in fat-1 mice.

### Lipidomics of endometriotic lesions in fat-1 and wild type mice

**Figure 2:**
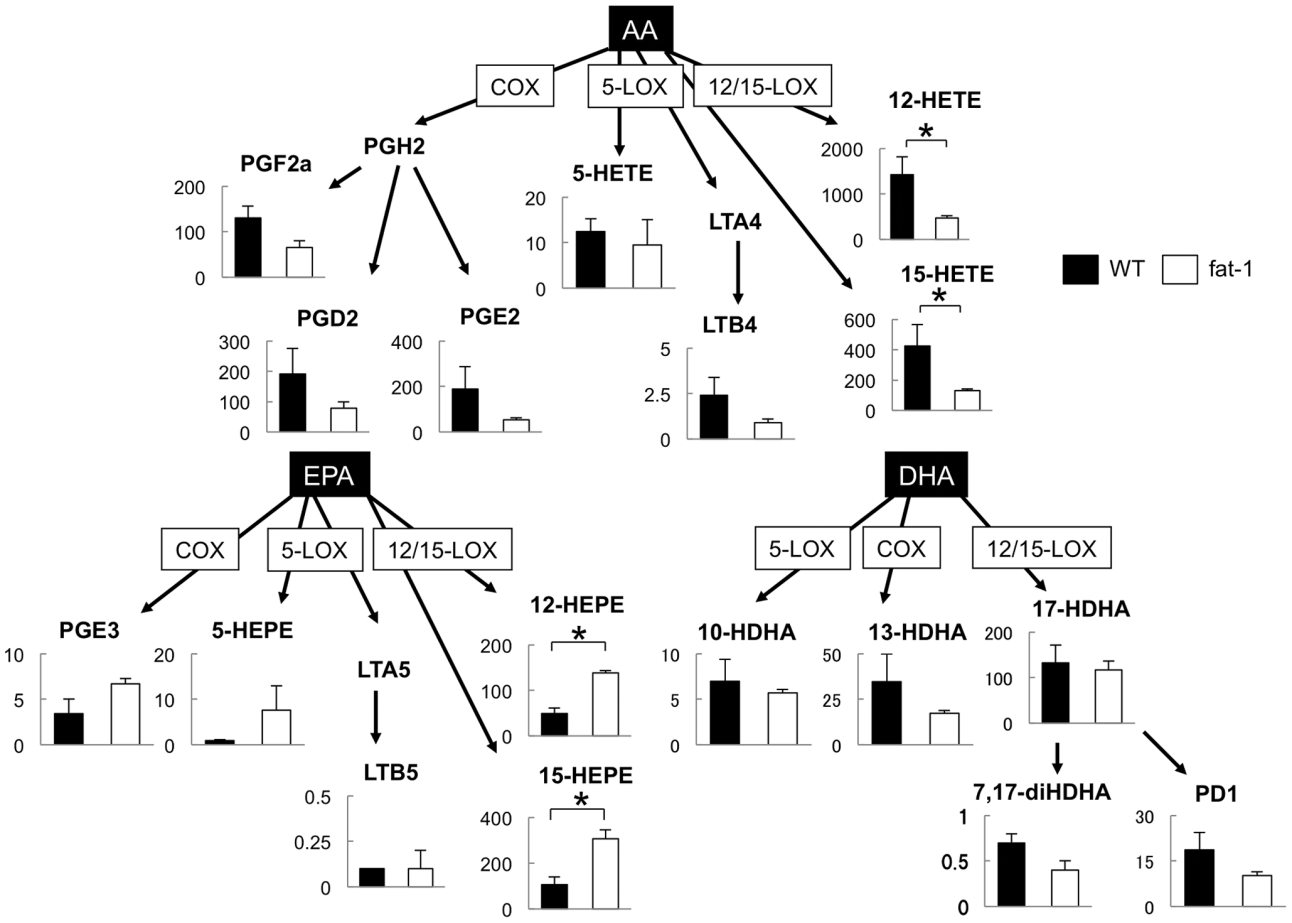
Lipid mediator analyses of endometriotic lesions: wild type vs. fat-1 mice. Endometriotic lesions obtained from fat-1 (white) or wild type (WT: black) mice were assessed by lipidomic analyses (n = 3 in each group). The main products of AA-, EPA- and DHA-derived mediators are indicated. Y axis denotes the amount of each lipid mediator (pg/g sample). Mean values with standard deviations are presented. Asterisks indicate those comparisons (fat-1 vs. wild type mice) with statistical significance (p<0.05).

**Figure 3:**
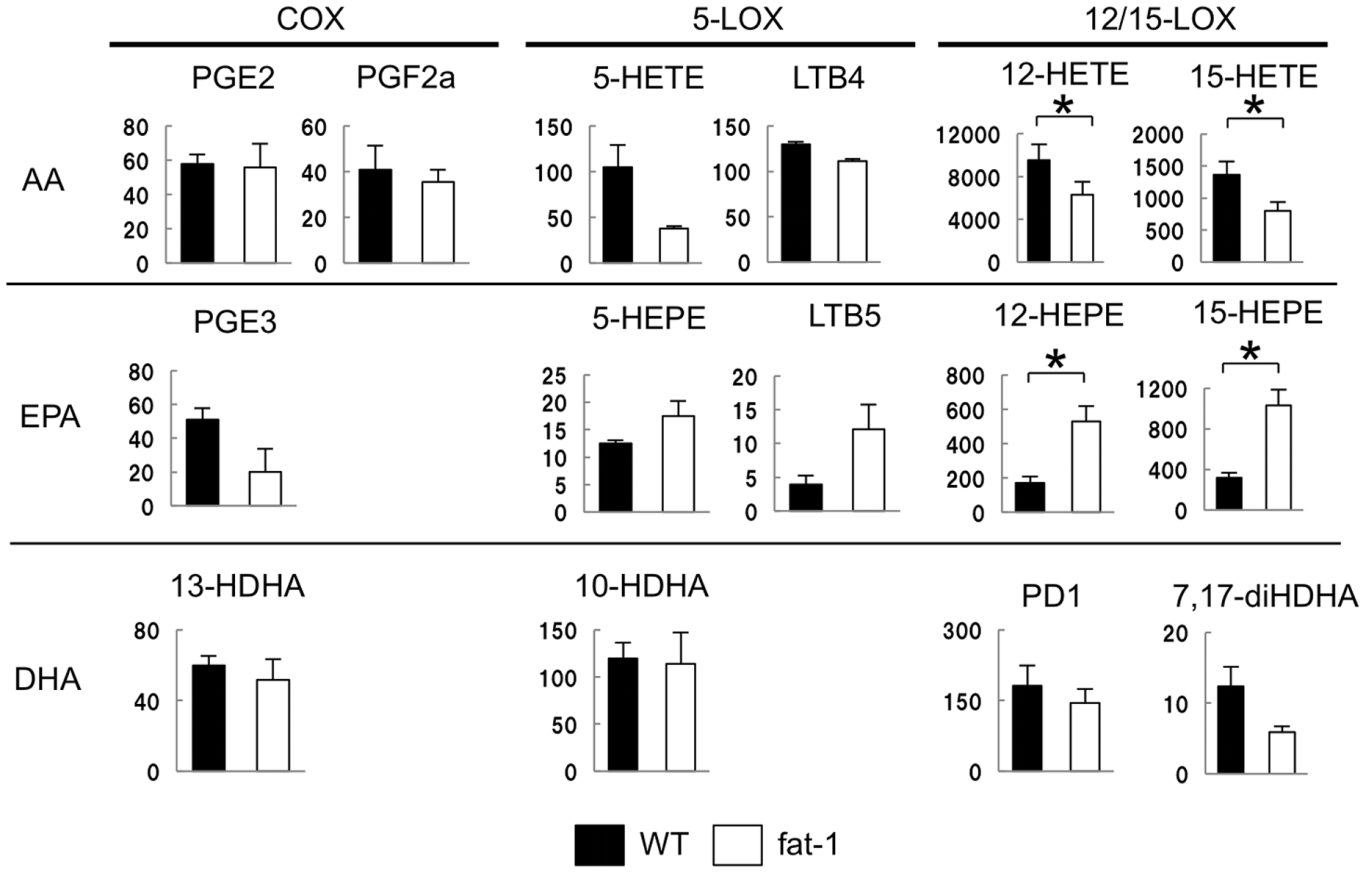
Lipid mediator analyses of peritoneal fluids: wild type vs. fat-1 mice. Peritoneal exudates of mice developing endometriotic lesions were collected by washing with saline. The peritoneal fluids obtained from the fat-1 (white) or wild type (WT: black) mice were analyzed as shown in the Fig. 3 (n = 3 in each group). The main products of AA-, EPA- and DHA-derived mediators are indicated. Y axis donates the amount of each lipid mediator (pg/g sample). Mean values with standard deviations are presented. Asterisks indicate those comparisons (fat-1 vs. wild type mice) with statistical significance (p<0.05).

Fat-1 mice showed a decreased number and weight of cystic endometriotic lesions suggesting that increased omega-3 PUFAs are associated with the suppression of endometriosis. To address the mechanisms involved in this suppressive effect, LC-MS/MS-based lipidomic analyses were performed to monitor lipid mediators derived from omega-3 as well as omega-6 PUFAs [26]. First, the endometriotic lesion was examined by lipidomic analyses and a direct comparison was made for PUFA metabolites between fat-1 and wild type mice (n = 3 in each group) (Fig. 2). A whole series of EPA metabolite was significantly increased in fat-1 mice compared to the wild type. Among the EPA metabolites, most difference in 12/15-hydroxyeicosapentaenoic acids (HEPE) levels was observed (Fig. 2). As for DHA, there was no metabolite showing a significant difference between fat-1 and wild type mice (Fig. 2). In contrast, AA metabolites in fat-1 mice were generally lower than those in the wild type mice (Fig. 2). Next, peritoneal exudates were collected from the endometriosis-present peritoneal cavity of fat-1 or wild type mice and were assessed for PUFA metabolite profiles. Again, there was no difference in the amounts of DHA metabolites between fat-1 and wild type mice (data not shown). The main products derived from EPA and AA in peritoneal cavity were shown in Fig. 3. Peritoneal fluids were abundant in 12/15-HEPE in EPA metabolites and 12/15-hydroxyeicosatetraenoic acids (HETE) in AA metabolites. The amounts of 12/15-HEPE in fat-1 mice were significantly greater than that in wild type mice, as shown in endometriotic lesions (Fig. 3, center panel). Among AA metabolites, a significant difference in the amounts of 12/15-HETE between fat-1 and wild type mice was shown (Fig. 3, upper panel). These were the same findings as those shown in endometriotic lesions. Taken together, the increased amount of 12/15-HEPE was characterized markedly in both the endometriotic lesions and peritoneal cells of fat-1 mice.

### Endometriosis in the 12/15-LOX-KO mice

**Figure 4:**
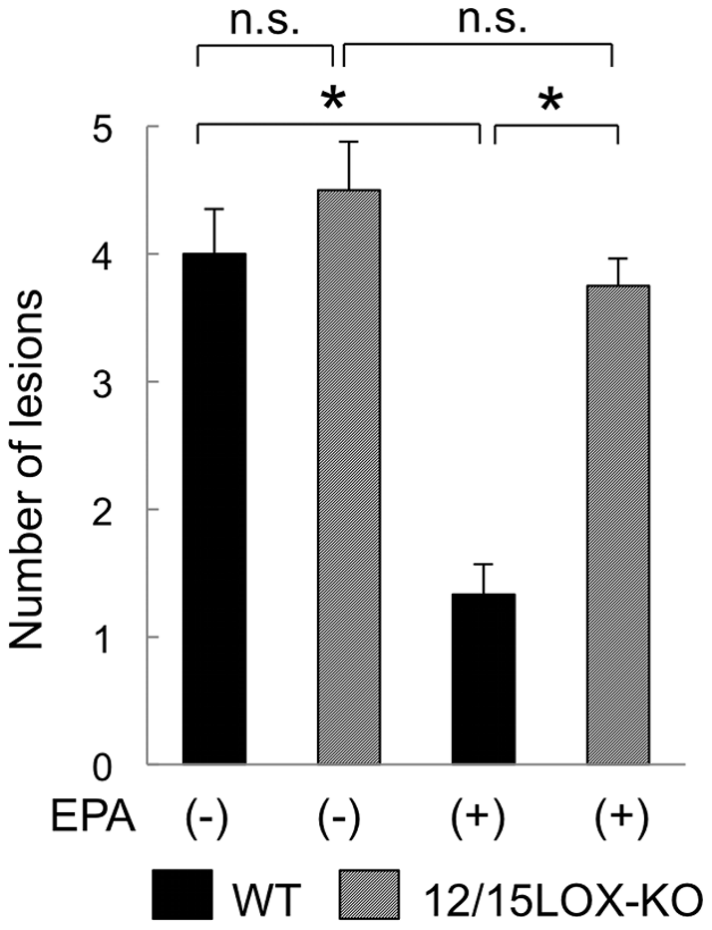
Comparison of the number of endometriotic lesions between wild type and 12/15-LOX-KO mice with or without EPA administration. Endometriotic lesions were generated in wild type (WT: black) and 12/15-LOX-KO (stripe) mice with or without oral administration of 5% EPA ethyl ester. The number of endometriotic lesions was compared between the wild type and 12/15-LOX-KO mice with or without EPA oral administration (n = 4 in each group). Mean values with standard deviations are presented. Asterisks indicate those comparisons with statistical significance (p<0.05).

EPA-derived 12/15-HEPE was higher and AA-derived 12/15-HETE was lower in the endometriotic lesions of fat-1 mice than of those in wild type mice. Since both 12/15-HEPE and 12/15-HETE are converted by 12/15-LOX, 12/15-LOX-related mediators may play a role in protection against the development of peritoneal endometriotic lesions. Then 12/15-LOX-KO and wild type mice were administered EPA orally to address the effect of 12/15-LOX-related mediators on endometriotic lesions by comparing the number of the lesions with that of wild type mice. Endometriotic lesions were generated in wild type and 12/15-LOX-KO mice with or without EPA administration (n = 4 in each group) (Fig. 4). EPA administration decreased significantly the number of endometriotic lesions in wild type mice. However, the suppressive effect by EPA administration on the development of endometriotic lesions was cancelled in 12/15-LOX-KO mice. In 12/15-LOX-KO mice with or without EPA administration, the number of endometriotic lesions was the same level as that of wild type mice with no administration.

**Figure 5:**
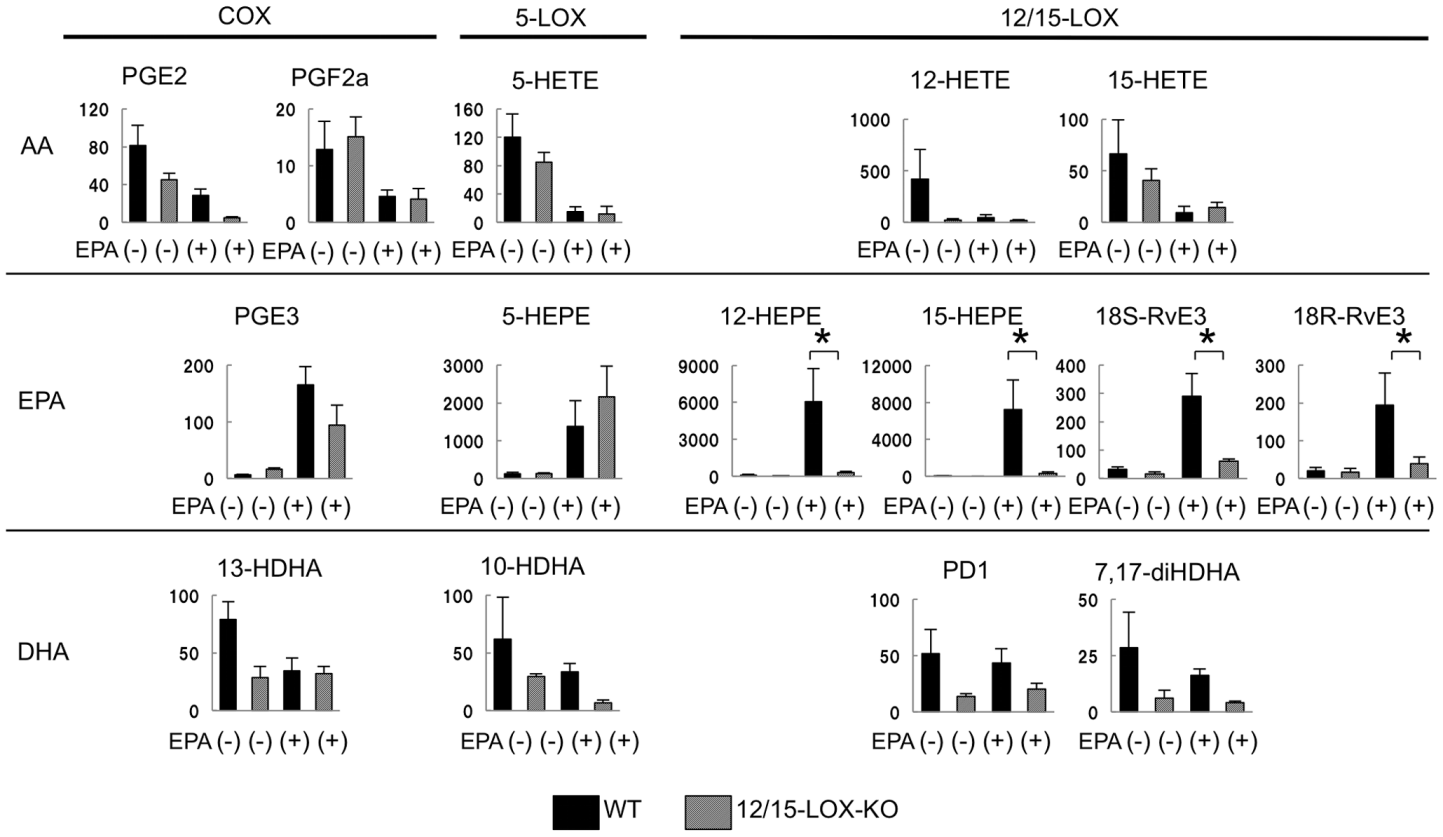
Lipid mediator analyses of peritoneal fluids from wild type or 12/15-LOX-KO mice with or without EPA administration. Peritoneal exudates of mice developing endometriotic lesions were collected by washing with saline. The peritoneal fluids obtained from mice as shown in Fig.(n = 3 in each group). The main products of AA-, EPA- and DHA-derived mediators were indicated. Y axis denotes the amount of each lipid mediator (pg/g sample). Mean values with standard deviations are presented. Asterisks indicate those comparisons (wild type vs. 12/15-LOX-KO mice) with significance (p<0.05).

Then we examined amounts of lipid mediators derived from omega-3 as well as omega-6 PUFAs in the peritoneal fluids obtained from wild type and 12/15-LOX-KO mice with or without EPA administration (n = 3 in each group) (Fig. 5). In wild type mice with EPA administration, all EPA metabolites were significantly increased compared with wild type mice without EPA administration. In comparison between wild type and 12/15-LOX-KO mice with EPA administration, 12/15-HEPE was significantly decreased in 12/15-LOX KO mice while PGE3 and 5-HEPE were almost equal amount between wild type and 12/15-LOX-KO mice. Interestingly, EPA-derived bioactive mediators, 18S/R-resolvin E3 (RvE3) which is biosynthesized from 18-HEPE by 12/15-LOX [27], was increased in peritoneal fluids of wild type mice after EPA administration (Fig. 5, center panel). Again, the increase of RvE3 was canceled in 12/15-LOX-KO mice. The other E-series resolvins, RvE1 and RvE2, were negligible amounts in both mice (data not shown). As for AA metabolites, there was no difference in amounts of AA metabolites between wild type and 12/15-LOX-KO mice after EPA administration.

### Comparison of expression profiles in endometriotic lesions between fat-1 and wild type mice

In humans, the symptoms of endometriosis are thought to result from an excessive inflammatory environment within the peritoneal cavity. Elevated numbers of activated immune cells, including macrophages, are involved in the production of inflammatory cytokines. The expression profiles of endometriotic lesions in our mice model were assessed using cDNA microarrays. A comparison was made for the expression of the main cytokines between fat-1 and wild type mice and the ratio of fat-1/wild type for each cytokine was indicated in Table 1 (n = 3 in each group). Among the cytokines examined, the IL-6 was the only one which was reduced of a ratio greater than 0.5. Indeed, IL-6 expression in endometriotic lesions of fat-1 mice was one fifth lower than that in wild type mice.

**Table 1:** Comparison of the cytokine/chemokine profiles of peritoneal endometriotic lesions between fat-1 and wild type (WT) mice (n = 3 in each group).

Cytokines	fat-1/WT ratio
**IL-6**	**0.2**
TNF-α	0.6
TGF-β1	0.8
TGF-β2	0.6
IL-1b	0.7
IL-4	0.5
IL-10	0.5
IL-12b	1.0
IL-17a	0.9

### Suppression of IL-6 mRNA production in peritoneal macrophages in fat-1 mice

**Figure 6:**
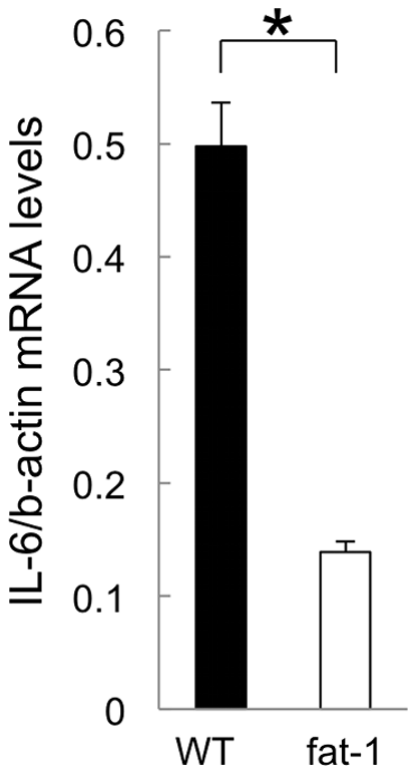
IL-6 production of peritoneal macrophages derived from fat-1 and wild type mice. Peritoneal fluids were collected from the fat-1 (white) and wild type (WT: black) mice as shown in Fig. 4 (n = 5 in each group). Among them, peritoneal macrophages were isolated by CD11b-beads selection. IL-6 mRNA levels in isolated macrophages were measured by RT-quantitative PCR. IL-6 mRNA levels were normalized to β-actin. Mean values with standard deviations are presented. Asterisks indicate those comparisons (fat-1 vs. wild type mice) with statistical significance (p<0.05).

Peritoneal macrophages are one of the most important immune cells to play a role in the development and progression of endometriosis in the peritoneal cavity. Interestingly, peritoneal macrophages possess 12/15-LOX abundantly and, in turn, 12/15-LOX expressing cells are predominantly macrophages in the peritoneal cavity in mice, while circulating and myeloid monocytes do not express 12/15-LOX. We focused on peritoneal macrophages as a source of IL-6 and isolated macrophages from the peritoneal cavity of fat-1 and wild type mice by CD11b-positive selection. A comparison was made between the fat-1 and wild type mice for IL-6 mRNA expression in peritoneal macrophages (n = 5 in each group) (Fig. 6). IL-6 mRNA production in macrophages in fat-1 mice was significantly suppressed by approximately one fifth that of wild type mice.

## Discussion

Numerous studies utilize fat-1 mice to examine the effects of omega-3 PUFAs and the resultant anti-inflammatory mediators on retarding inflammatory disease development and/or progression to cancer. These models include LPS-induced hepatitis, dextrane sodium sulfate (DSS)-induced colitis, and chronic colitis-associated colon cancer [28]–[30]. Because it is possible to collect and analyze peritoneal fluid, modified mice are particularly suitable for intraperitoneal disease models. We find fat-1 mice very suitable as models of endometriosis, because endometriosis is an intraperitoneal disease. Fat-1 mice allow carefully controlled studies to be performed in the absence of potential confounding factors of diet. Here, fat-1 mice allowed us to investigate essential endogenously biosynthesized mediators to suppress endometriotic lesions.

In this mouse model used in our experiment, cystic lesions were formed as endometriotic lesions macroscopically. By counting the number of them, and then excised them from surrounding normal tissues, we could evaluate the suppressive effects on lesion formation. In the results of this study, we could observe a significant difference on lesion formation at the macroscopic level. But some problems still remain. We could not evaluate and excise exactly small and invisible lesions to the naked eye. For example, it is sometimes difficult to distinguish small non-cystic lesions and microscopic lesions from surrounding normal tissues. As a solution, an animal model using green fluorescence protein (GFP) mice may be useful in order to evaluate the small non-cystic and microscopic lesions [21]. In this study, we used two kinds of genetically modified mice, fat-1 and 12/15-LOX KO mice. Because of some technical problems of mating, crossing, and so on, it was not possible to use GFP mice in the same way as the previous report in this study. But in our further research, the introduction of GFP mice is expected to be useful for the evaluation model of the effects on lesion formation.

Several previous studies have experimentally demonstrated the suppressive effects of omega-3 PUFAs on endometriosis. Two studies have used endometriosis animal models in which animals were fed EPA [31] or fish oil [20] and another used human endometrial stromal cells obtained from endometriosis patients that were incubated with PUFAs *in vitro* [32]. All previous studies on this field were not able to strictly address the mechanism or lipid mediator by which endometriosis was suppressed. It is difficult to make dietary components identical in both quality and quantity when feeding animals an omega-3 PUFA-rich diet. In animals administered purified EPA, the enrichment of EPA in the animal organs should be transient. These methodologic limitations may hamper identification of the biosynthesized mediators responsible for the suppressive function against endometriosis.

When n-6/n-3 PUFAs levels become low, they prevent the generation of pro-inflammatory eicosanoids from AA and convert n-3 PUFAs into anti-inflammatory metabolites. As cellular level mechanisms of anti-inflammatory effects, increased levels of n-3 PUFAs and their functional EPA or DHA-derived metabolites, such as resolvins and protectins, help resolve inflammation mostly through reductions, in neutrophils trafficking and upregulation of macrophage-mediated removal of apoptotic cells [1]. For example, when EPA-derived RvE1 interacts with BLT1, which is a receptor expressed on neutrophils, pro-inflammatory signals from LTB4 are attenuated and LTB4-stimulated migration of neutrophils to inflammatory sites is suppressed [5]. Moreover, when RvE1 interacts with ChemR23, which is a membrane receptor expressed on macrophages, phagocytosis and transport to lymph nodes is enhanced and the excessive activation of NF-κB is attenuated [4]. In patients with endometriosis, NF-κB expression is known to be increased. NF-κB inhibitors have attracted attention as a novel treatment in recent years [33], [34]. These mechanisms indicated that the suppressive effects of n-3 PUFAs and metabolites on endometriosis may be related to attenuation of excessive NF-κB activation.

In this study, global PUFA metabolite profiles in endometriotic lesions and peritoneal cells were characterized by lipidomic analyses between fat-1 and wild type mice. Analyses revealed that 12/15-HEPE were converted mainly from EPA in peritoneal endometriosis and these amounts were approximately three-fold larger in fat-1 mice than that in wild type mice. Increased 12/15-HEPE amounts were observed similarly in both endometriotic lesions and peritoneal exudates. This allowed us to focus on 12/15-LOX-related mediator as a possible lipid mediator to suppress endometriosis; 12/15-LOX-KO mice were then utilized to address their suppressive effect on peritoneal endometriotic lesions. In wild type mice, EPA administration protected against the development of endometriotic lesions, consistent with the results of a previous study [31]. This suggested EPA and/or any EPA-derived mediators exhibit suppressive effect on the endometriotic lesions in fat-1 mice. Interestingly, the suppressive effect was canceled in 12/15-LOX-KO mice although the mice were administered EPA, suggesting that EPA itself may not have a central effect on the development of endometriotic lesion. Our lipid mediator analyses demonstrated that amounts of 12/15-LOX-related metabolites such as 12/15-HEPE and RVE3 in wild type mice were much larger than those in 12/15-LOX-KO mice after EPA administration. RVE3 is recently identified as a novel EPA-derived anti-inflammatory bioactive mediator which is biosythesized from 18-HEPE via 12/15-LOX pathway [27]. Taken together, endometriotic lesions seem to be suppressed in a manner dependent on 12/15-LOX pathway. EPA-derived 12/15-LOX-related mediators may play a role in the protection effect in this model.

Interestingly, more than 95% of murine peritoneal macrophages express 12/15-LOX [35]. In turn, the predominant population expressing 12/15-LOX is resident peritoneal macrophages in mice [36]. Peritoneal macrophages are increased in number and more activated in patients with endometriosis [37], [38] and are one of the major sources for inflammatory cytokines in the peritoneal cavity. Therefore, peritoneal macrophages are thought to be involved in the development of endometriosis. The increased amounts of 12/15-LOX-related EPA metabolites in the peritoneal exudates of fat-1 mice may originate from peritoneal macrophages enriched in omega-3 PUFA. The anti-inflammatory effect of EPA metabolites may inhibit the development of peritoneal endometriotic lesions. The decreased IL-6 mRNA levels in the peritoneal cells of fat-1 mice seem to reflect anti-inflammatory actions. One study demonstrated that there were 1.7-fold more peritoneal macrophages in 12/15-LOX-KO mice than that in wild type mice and that the 12/15-LOX pathway exerted negative effects on monocyte/macrophage migration into the peritoneal cavity [39]. This decrease may be favorable for protection against the development of endometriotic lesions in 12/15-LOX-KO mice.

In this study, we showed the suppressive effect of omega-3 PUFAs in the mouse endometriosis model by making full use of two types of genetically modified mice: fat-1 and 12/15-LOX KO mice. To take sufficient amounts of omega-3 PUFAs, we need to eat a lot of fish and fish oil or EPA supplements. However, there are limitations to the amount of sufficient omega-3 PUFAs intake, so we think it is desirable that we identify more effective metabolites than omega-3 PUFAs themselves. We revealed the function of key metabolites in the suppressive mechanism by using lipidomic analysis. Further research will provide more insight into the effects of omega-3 PUFAs and possibly lead to the treatment for endometriosis involving novel anti-inflammatory mediators.
